# Clarifying the Size–Frequency Relationship in Clownfish Acoustic Signals

**DOI:** 10.1002/ece3.73176

**Published:** 2026-02-25

**Authors:** Eric Parmentier

**Affiliations:** ^1^ Laboratory of Functional and Evolutionary Morphology, Freshwater and Oceanic Science Unit of Research University of Liège Liège Belgium

**Keywords:** acoustic, communication, Pomacentridae, sonic

## Abstract

Body size is the principal determinant of acoustic variation in anemonefish, reflecting both the mechanics of sound production and the size‐based structure of their social hierarchies. In *Amphiprion percula* , the absence of a reported size‐frequency relationship has led to the interpretation that small acoustic differences are rank‐specific. We show that this outcome stems from analytical choices that obscure natural size variation, including pooling individuals across groups and removing size–rank covariance. Because behavioural categories correspond to distinct size classes, morphology must be explicitly accounted for.

In a recent study on acoustic communication in anemonefish, Yllan and Rueger (2025) provide valuable field observations on the vocal behaviour of *Amphiprion percula* , offering rare in situ data on sound production within natural social groups. However, several aspects of their interpretation warrant clarification. Most importantly, the authors report no significant effect of body size on dominant or peak frequency after statistically removing the natural covariation between size and social rank. This approach effectively eliminates the biological signal expected in clownfish, whose hierarchies and acoustic scaling are tightly size‐dependent. Here, ‘body size’ refers to linear body dimensions (standard length), which directly constrain the mechanics of sound production. As a result, the absence of a size–frequency relationship in their analysis reflects a methodological artefact, not a deviation from established patterns. Existing studies on clownfish species consistently demonstrate a strong negative relationship between body size and dominant frequency (Colleye et al. 2009; Colleye and Parmentier 2012). This pattern is central to our understanding of agonistic communication and hierarchy maintenance in *Amphiprion*. Interpreting small acoustic differences as rank‐specific therefore lacks support from the biomechanics of sound production. Here, I revisit this issue using only published evidence to explain why the conclusions drawn by the authors (Yllan and Rueger 2025) are unlikely to reflect the natural biology of *Amphiprion*.

Clownfish produce aggressive and submissive sounds during agonistic interactions toward con‐ and heterospecifics (Allen 1972; Colleye and Parmentier 2012; Schneider 1964). Aggressive and submissive sounds in clownfish differ clearly in structure, temporal properties, amplitude, and behavioural context. Aggressive sounds (threatening and fighting sounds) are produced by dominant individuals during charges and chases (Chen and Mok 1988; Parmentier et al. 2005; Takemura 1983); they consist of single‐pulse units, emitted alone or in series, and display longer pulse durations and pulse periods. In contrast, submissive sounds are emitted exclusively by subordinate individuals during head‐shaking displays. They are always multi‐pulsed, never consisting of a single pulse, and show much shorter pulse periods and shorter pulse durations. In *Amphiprion frenatus* , for example, pulse periods average 12 ms versus 106 ms in aggressive sounds, and pulse durations 8 ms versus 14 ms, respectively (Colleye and Parmentier 2012; Parmentier and Lecchini 2022).

Aggressive sounds arise from rapid jaw closure (snapping) and tooth collisions. This jaw slam induces vibrations of the skeleton and rib cage, which in turn move the closely associated swim bladder wall, acting like a loudspeaker membrane and radiating the sound (Olivier et al. 2015; Parmentier et al. 2007). What is firmly established is that aggressive sounds involve vibration of structural units, but not resonance of bone material itself, and these vibrating structures necessarily scale with body size (Colleye et al. 2012). No component of this mechanism permits modulation of oscillation rate independently of the size of the vibrating structures. This is fully consistent with the observation that dominant frequency scales only with body size in all clownfish species studied to date. This mechanical reality is consistent across fishes that produce short, pulse‐like sounds through mechanisms not involving rapid sonic muscles (Parmentier and Fine 2016). The relationship between peak frequency and size has been shown in other pomacentrids (Myrberg et al. 1993) and in taxa such as cichlids (Amorim et al. 2003; Bertucci et al. 2015), triggerfish (Raick et al. 2017) or gobiids (Malavasi et al. 2003). The mechanism of submissive sounds, however, remains unknown and cannot be assumed to operate identically to aggressive sound production.

It results in *Amphiprion* species, dominant frequency and pulse duration are tightly constrained by morphology. In *A. akallopisos* , dominant frequency correlates strongly and negatively with standard length (*r* = −0.97), with larger individuals producing lower dominant frequencies, while pulse duration shows a positive correlation with body size (Colleye et al. 2009). Similar size‐dependent trends have been demonstrated in *A. frenatus* across a 44–112 mm range (Colleye and Parmentier 2012). Moreover, across 15 clownfish species, the frequency–size relationship follows an identical slope, indicating that the scaling mechanism is conserved throughout the genus (Colleye et al. 2011). These results strongly support the idea that acoustic features primarily reflect morphology.

Clownfish social organisation further reinforces this expectation. Social hierarchies rely on strict size ratios: adjacent ranks differ on average by a factor of ~1.26, although some size overlap between ranks does occur (P. Buston 2003; Buston and Cant 2006; Colleye et al. 2016). As a result, individuals of identical size may occupy different ranks depending on the colony. Conversely, individuals of the same rank do not necessarily share the same absolute body size, since both males and females may reach sexual maturity at slightly different sizes. What structures the hierarchy is therefore not the absolute size associated with a given rank, but the relative size differences between adjacent group members (P. Buston 2003; Buston and Cant 2006). Acoustic signals are thought to contribute to the maintenance of this hierarchy by reinforcing perceived size differences (Colleye and Parmentier 2012) and preventing escalated conflict (P. Buston 2003, 2004; Fricke 1979). For this system to function, acoustic cues must reliably encode body size. If two individuals of similar size but different rank produced different dominant frequencies, the honesty of the signal would be compromised and assessment would become unreliable. Playback experiments are therefore ultimately required to demonstrate that clownfish are able to discriminate conspecifics on the basis of acoustic cues alone. To date, such experiments are still lacking in *Amphiprion*. Nevertheless, two independent lines of evidence provide important indications that acoustic discrimination based on size‐related frequency differences is biologically plausible in this group. First, auditory sensitivity measurements in juvenile specimens (31–55 mm total length) of *Amphiprion frenatus* , *A. ocellaris* and *A. clarkii* show that their best hearing sensitivity lies below the dominant frequency of their own calls, but close to the dominant frequencies produced by larger individuals (Parmentier et al. 2009). This pattern suggests that juveniles may be particularly tuned to detecting sounds emitted by adults, consistent with a functional role of acoustic cues in social interactions and spatial localisation of dominant group members. Second, in another pomacentrid species, the bicolor damselfish *Pomacentrus partitus* , playback experiments have demonstrated that females use male courtship sounds to locate nests. Importantly, females were able to discriminate between males differing by as little as 4 mm in body size, based solely on differences in dominant frequency, approximately 710 Hz versus 780 Hz (Myrberg et al. 1986). This result provides direct experimental evidence that small spectral differences linked to body size can be perceptually meaningful in pomacentrid fishes. Together, these findings strongly support the hypothesis that size‐related acoustic parameters are functionally relevant in social communication within Pomacentridae. While targeted playback experiments remain necessary to directly test acoustic size discrimination in clownfish, existing auditory and behavioural data are fully consistent with the idea that dominant frequency constitutes an honest and biologically interpretable cue of body size.

Consequently, using social rank as a categorical predictor risks obscuring underlying morphological variation. This issue is amplified by the fact that all individuals from all social groups were analysed together, without explicitly modelling the size structure or hierarchy specific to each group. In clownfish, rank categories do not guarantee size homogeneity (either within or across groups) and statistically centring body size further removes the natural covariance between rank and morphology. From a functional perspective, comparisons of acoustic features should therefore be based primarily on body size rather than rank, since only body size captures the mechanical constraints governing sound production. This distinction is essential for determining whether observed acoustic differences reflect true size‐related scaling or methodological artefacts arising from hierarchical classification and pooled analyses. Furthermore, directly comparing acoustic parameters across behavioural categories may introduce a substantial methodological bias. In size‐based hierarchies such as those of anemonefish, submissive behaviours are almost exclusively performed by the smallest individuals in the group (Colleye and Parmentier 2012), whereas aggressive behaviours, whether directed at conspecifics or heterospecifics, are typically produced by the largest individuals. As a result, each behavioural category is intrinsically associated with a distinct size class, which mechanically shifts the mean acoustic values attributed to each behaviour.

To robustly assess differences that are genuinely linked to behavioural context, it would be preferable to normalise acoustic parameters by body size, as recommended in previous studies (Banse et al. 2024; Colleye and Parmentier 2012; Raick et al. 2017). Without such correction, the observed variations are likely to reflect mainly the effect of body size rather than the behavioural context itself, which may in turn affect the functional interpretation of the acoustic signals assigned to each behaviour.

Taken together, published data show that clownfish acoustic signals primarily encode body size, a property central to the establishment and maintenance of social hierarchies. Because these hierarchies rely on stable size ratios, substantial rank‐specific variation in dominant or peak frequency would be inconsistent with the known mechanics of sound production. The absence of a size–frequency relationship in *Amphiprion percula* reported by Yllan and Rueger (2025) is therefore unexpected in light of established size‐related scaling and is likely influenced by aspects of analytical design, such as pooling individuals across groups and statistically removing the natural covariance between size and rank, which can obscure biological variation. Before drawing behavioural conclusions, acoustic parameters should be re‐evaluated with analyses that explicitly incorporate precise body‐size measurements and treat size and rank as distinct sources of variation. Only then can the functional significance of acoustic variation in *Amphiprion percula* be reliably assessed.

## Author Contributions

**Eric Parmentier:** conceptualization (lead), writing – original draft (lead), writing – review and editing (lead).

## Conflicts of Interest

The author declares no conflicts of interest.

## Data Availability

The author has nothing to report.

## References

[ece373176-bib-0001] Allen, G. R. 1972. The Anemonefishes: Their Classification and Biology. Vol. 288. T.F.H. Publications.

[ece373176-bib-0002] Amorim, M. C. P. , P. J. Fonseca , and V. C. Almada . 2003. “Sound Production During Courtship and Spawning of *Oreochromis mossambicus*: Male—Female and Male—Male Interactions.” Journal of Fish Biology 62: 658–672.

[ece373176-bib-0003] Banse, M. , E. Bertimes , D. Lecchini , T. J. Donaldson , F. Bertucci , and E. Parmentier . 2024. “Sounds as Taxonomic Indicators in Holocentrid Fishes.” npj Biodiversity 3: 33. 39501023 10.1038/s44185-024-00064-4 PMC11538288

[ece373176-bib-0004] Bertucci, F. , P. Lejeune , J. Payrot , and E. Parmentier . 2015. “Sound Production by Dusky Grouper *Epinephelus marginatus* at Spawning Aggregation Sites.” Journal of Fish Biology 87: 400–421. 26177857 10.1111/jfb.12733

[ece373176-bib-0005] Buston, P. 2003. “Size and Growth Modification in Clownfish.” Nature 424: 145–146. 12853944 10.1038/424145a

[ece373176-bib-0006] Buston, P. M. 2004. “Territory Inheritance in Clownfish.” Proceedings of the Royal Society of London. Series B: Biological Sciences 271: S252–S254. 10.1098/rsbl.2003.0156 PMC1810038 15252999

[ece373176-bib-0007] Buston, P. M. , and M. a. Cant . 2006. “A New Perspective on Size Hierarchies in Nature: Patterns, Causes, and Consequences.” Oecologia 149: 362–372. 16794835 10.1007/s00442-006-0442-z

[ece373176-bib-0008] Chen, K.‐C. , and H.‐K. Mok . 1988. “Sound Production in the Anemonfishes, *Amphiprion clarkii* and *A. frenatus* (Pomacentridae), in Captivity.” Japanese Journal of Ichthyology 35: 90–97.

[ece373176-bib-0009] Colleye, O. , B. Frederich , P. Vandewalle , M. Casadevall , and E. Parmentier . 2009. “Agonistic Sounds in the Skunk Clownfish *Amphiprion akallopisos* : Size‐Related Variation in Acoustic Features.” Journal of Fish Biology 75: 908–916. 20738587 10.1111/j.1095-8649.2009.02316.x

[ece373176-bib-0010] Colleye, O. , E. Iwata , and E. Parmentier . 2016. Clownfishes. Edited by B. Frédérich and É. Parmentier. CRC Press, Taylor & Francis.

[ece373176-bib-0011] Colleye, O. , M. Nakamura , B. Frederich , and E. Parmentier . 2012. “Further Insight Into the Sound‐Producing Mechanism of Clownfishes: What Structure Is Involved in Sound Radiation?” Journal of Experimental Biology 215: 2192–2202. 22675179 10.1242/jeb.067124

[ece373176-bib-0012] Colleye, O. , and E. Parmentier . 2012. “Overview on the Diversity of Sounds Produced by Clownfishes (Pomacentridae): Importance of Acoustic Signals in Their Peculiar Way of Life.” PLoS One 7: e49179. 23145114 10.1371/journal.pone.0049179 PMC3492317

[ece373176-bib-0013] Colleye, O. , P. Vandewalle , D. Lanterbecq , D. Lecchini , and E. Parmentier . 2011. “Interspecific Variation of Calls in Clownfishes: Degree of Similarity in Closely Related Species.” BMC Evolutionary Biology 11: 365. 22182416 10.1186/1471-2148-11-365 PMC3282713

[ece373176-bib-0014] Fricke, H. W. 1979. “Mating System, Resource Defense and Sex Change in the Anemonefish *Amphiprion akallopisos* .” Zeitschrift für Tierpsychologie 50: 313–326.

[ece373176-bib-0015] Malavasi, S. , P. Torricelli , M. Lugli , F. Pravoni , and D. Mainardi . 2003. “Male Courtship Sounds in a Teleost With Alternative Reproductive Tactics, the Grass Goby, *Zosterisessor ophiocephalus* .” Environmental Biology of Fishes 66: 231–236.

[ece373176-bib-0016] Myrberg, A. A. J. , S. J. Ha , and M. J. Shamblott . 1993. “The Sounds of Bicolor Damselfish (*Pomacentrus partitus*): Predictors of Body Size and a Spectral Basis for Individual Recognition and Assessment.” Journal of the Acoustical Society of America 94: 3067–3070.

[ece373176-bib-0017] Myrberg, A. A. J. , M. Mohler , and J. Catala . 1986. “Sound Production by Males of a Coral Reef Fish (*Pomacentrus partitus*): Its Significance to Females.” Animal Behaviour 34: 913–923.

[ece373176-bib-0018] Olivier, D. , B. Frédérich , A. Herrel , and E. Parmentier . 2015. “A Morphological Novelty for Feeding and Sound Production in the Yellowtail Clownfish.” Journal of Experimental Zoology Part A: Ecological Genetics and Physiology 323: 227–238. 25777151 10.1002/jez.1907

[ece373176-bib-0019] Parmentier, E. , O. Colleye , M. L. Fine , B. Frédérich , P. Vandewalle , and A. Herrel . 2007. “Sound Production in the Clownfish *Amphiprion clarkii* .” Science 316: 1006. 17510359 10.1126/science.1139753

[ece373176-bib-0020] Parmentier, E. , O. Colleye , and D. Mann . 2009. “Hearing Ability in Three Clownfish Species.” Journal of Experimental Biology 212: 2023–2026. 19525428 10.1242/jeb.030270

[ece373176-bib-0021] Parmentier, E. , and M. L. Fine . 2016. “Fish Sound Production: Insights.” In Vertebrate Sound Production and Acoustic Communication, edited by R. Suthers , F. Tecumseh , A. N. Popper , and R. R. Fay , 19–49. Springer.

[ece373176-bib-0022] Parmentier, E. , J. P. Lagardère , P. Vandewalle , and M. L. Fine . 2005. “Geographical Variation in Sound Production in the Anemonefish *Amphiprion akallopisos* .” Proceedings of the Royal Society B: Biological Sciences 272: 1697–1703. 10.1098/rspb.2005.3146 PMC1559856 16087425

[ece373176-bib-0023] Parmentier, É. , and D. Lecchini . 2022. “Sound Communication.” In Evolution, Development and Ecology of Anemonefishes: Model Organisms for Marine Science, edited by V. Laudet and T. Ravasi , 95–102. CRC Press, Taylor & Francis.

[ece373176-bib-0024] Raick, X. , D. Lecchini , L. Kéver , O. Colleye , F. Bertucci , and É. Parmentier . 2017. “Sound Production Mechanism in Triggerfish (Balistidae): A Synapomorphy.” Journal of Experimental Biology 220: 186–193. 29170259 10.1242/jeb.168948

[ece373176-bib-0025] Schneider, H. 1964. “Bioakustische Untersuchungen an Anemonenfischen der Gattung *Amphiprion* (Pisces).” Zeitschrift für Morphologie und Ökologie der Tiere 53: 453–474.

[ece373176-bib-0026] Takemura, A. 1983. “Studies on the Underwater Sound—VIII. Acoustical Behavior of Clownfishes (*Amphiprion* spp.).” Bulletin of the Faculty of Fisheries. Nagasaki University 54: 21–27.

[ece373176-bib-0027] Yllan, L. , and T. Rueger . 2025. “‘What Does the Anemonefish Say?’: Investigating *Amphiprion percula*'s Acoustic Behaviour.” Ecology and Evolution 15: e72479. 41245429 10.1002/ece3.72479 PMC12617267

